# African Programme for Onchocerciasis Control 1995–2015: Updated Health Impact Estimates Based on New Disability Weights

**DOI:** 10.1371/journal.pntd.0002759

**Published:** 2014-06-05

**Authors:** Luc E. Coffeng, Wilma A. Stolk, Honorat G. M. Zouré, J. Lennert Veerman, Koffi B. Agblewonu, Michele E. Murdoch, Mounkaila Noma, Grace Fobi, Jan Hendrik Richardus, Donald A. P. Bundy, Dik Habbema, Sake J. de Vlas, Uche V. Amazigo

**Affiliations:** 1 Department of Public Health, Erasmus MC, University Medical Center Rotterdam, Rotterdam, The Netherlands; 2 African Programme for Onchocerciasis Control, Ouagadougou, Burkina Faso; 3 School of Population Health, The University of Queensland, Herston, Australia; 4 Department of Dermatology, Watford General Hospital, Watford, United Kingdom; 5 Human Development Network, The World Bank, Washington, D.C., United States of America; 6 Independent Consultant, Enugu, Nigeria; Imperial College London, United Kingdom

## Viewpoint

Since 1995, the African Programme for Onchocerciasis Control (APOC) has coordinated mass treatment with ivermectin in 16 sub-Saharan countries (Angola, Burundi, Cameroon, Central African Republic, Chad, Congo, Democratic Republic of Congo, Equatorial Guinea, Ethiopia, Liberia, Malawi, Nigeria, North Sudan, South Sudan, Uganda, and the United Republic of Tanzania) with the aim to control morbidity due to infection with *Onchocerca volvulus*, a filarial nematode. Recently, we predicted trends in prevalence of infection, visual impairment, blindness, and troublesome itch due to onchocerciasis in APOC countries for the period 1995–2015, based on extensive data on pre-control infection levels, population coverage of ivermectin mass treatment, and the association between infection and morbidity [1]. We also estimated the associated health impact, expressed in disability-adjusted life years (DALYs). However, the estimated health impact was based on disability weights from the 2004 update of the Global Burden of Disease (GBD) study [2], which have been criticized for being based solely on the opinions of health professionals [3], [4]. The recently published GBD 2010 study addressed this criticism by providing updated disability weights based on household surveys in Bangladesh, Indonesia, Peru, and Tanzania, an open internet survey, and a telephone survey in the United States [5]. As a result of this population-based approach, the disability weights for visual impairment, blindness, and troublesome itch have changed considerably and should better reflect our ideas and beliefs as a society of what constitutes health. For future reference, we provide an updated estimate of the health impact of APOC activities, based on previously predicted trends in averted number of cases with infection and morbidity, but using updated disability weights for visual impairment, blindness, and troublesome itch.

Identical to previously used methods [1], we calculated the health impact of APOC for each year between 1995 and 2015, expressed in DALYs averted. The DALY metric is the sum of years of life lost (YLL) due to premature mortality (from blindness) and years lived in disability (YLD), weighted by a disability weight representing the loss of quality of life [5]. DALYs averted were calculated as the difference between two scenarios: a factual scenario in which APOC activities have taken place as documented, and a counterfactual scenario in which APOC activities have not taken place at all, effectively translating to 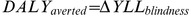 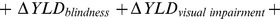 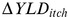. Here, 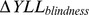 is the averted number of YLL related to premature mortality from blindness (as previously estimated [1]), and 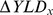 is the averted number of YLD due to symptom *x*. Averted YLD were calculated as 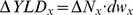
_,_ where 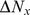 is the averted number of person-years of symptom *x* (i.e., difference in annually prevalent cases between the factual and counterfactual scenarios, as previously estimated [1]), and 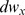 is the associated updated disability weight, derived from the GBD 2010 study [5].

Compared to previous disability weights [2], updated weights were considerably lower for visual impairment (0.033, previously 0.282) and blindness (0.195, previously 0.594), reflecting that the loss in quality of life because of these manifestations is considerably lower than previously assumed. On the contrary, the disability weight for troublesome itch has increased (0.108, previously 0.068). The disability weight for visual impairment represents “moderate visual impairment” in the GBD 2010 study. The updated disability weights do not include a category for itch alone. Hence the disability weight for troublesome itch was derived from a generic class of disability weights for “disfigurement with itch or pain.” This class consists of three severity levels, characterized as “causing some worry and discomfort” (disability weight 0.029), “a person having trouble concentrating and sleeping” (disability weight 0.187), and “causing a person to avoid social contact, feel worried, sleep poorly, and think about suicide” (disability weight 0.562). Based on original precontrol data from a previously published, multicountry study [6] (excluding data from Ghana and Cameroon, which were collected based on convenience sampling rather than household surveys), we assumed that onchocercal itch regularly causes insomnia in about half of the cases and, therefore, calculated YLD due to itch using the mean of the disability weights for the first two severity levels (0.108). We assumed that this disability weight also applies during ivermectin mass treatment, even though the fraction of insomniacs among cases of itch might decrease with repeated mass treatments (due to lower infection loads and consequent lower severity of itch). Unfortunately, previous studies on trends of onchocercal itch during ivermectin mass treatment do not report on insomnia [7], [8]. Therefore, if anything, we may be underestimating the impact of ivermectin mass treatment on the burden of itch (and the associated DALYs averted).

**Figure 1:**
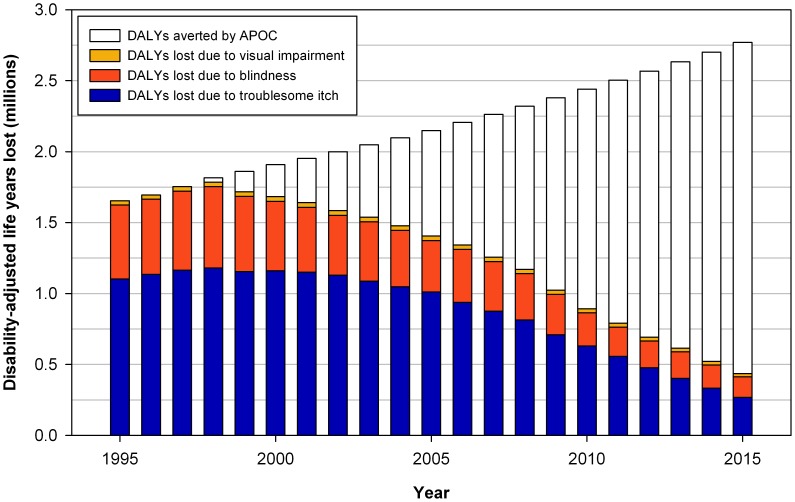
Disability-adjusted life years (DALYs) lost due to onchocerciasis from 1995 to 2015. The total height of the bars (colored plus blank) represents the estimated number of DALYs lost in a counterfactual scenario without ivermectin mass treatment (increasing trend due to population growth). The colored part of each bar represents the estimated actual number of DALYs lost (declining trend due to ivermectin mass treatment). The blank part of each bar therefore represents the annual number of DALYs averted by ivermectin mass treatment in the total APOC population.

Figure 1 illustrates trends in DALYs lost due to troublesome itch, visual impairment, and blindness, and DALYs averted by APOC. Table 1 gives more detailed information on the number of prevalent cases (according to the factual scenario) and DALYs lost and averted per year. For onchocercal visual impairment and blindness, the updated estimates of the averted burden turned out lower than the previous estimates. In contrast, for troublesome itch, the updated estimate of the burden averted turned out higher than the previous estimate. For visual impairment and troublesome itch, the difference between previous and updated estimates was proportional to the change in values of the associated disability weights. For blindness, however, this difference was not proportional, as the burden of blindness also included years of life lost due to premature mortality (which is exactly the same for previous and updated estimates).

**Table 1:** Population at risk, number of cases, and disability-adjusted life years lost and averted due to onchocerciasis in areas covered by APOC.

Year	Population size and number of cases of infection and disease in APOC areas (thousands)					Disability-adjusted life years lost (thousands)				Disability-adjusted life years averted (thousands)			
	Population (At risk of infection)	Infected^a^	Troublesome itch	Visual impairment	Blindness	Troublesome itch	Visual impairment	Blindness	Total	Troublesome itch	Visual impairment	Blindness	Total
1995	71,474	32,330	10,202	889	404	1,102	29	523	1,654	0	0	0	0
1996	73,310	33,209	10,499	910	410	1,134	30	530	1,694	0	0	0	0
1997	75,195	34,073	10,780	931	418	1,164	31	558	1,753	0	0	0	0
1998	77,132	34,951	10,925	957	427	1,180	32	573	1,785	9	0	21	30
1999	79,122	35,816	10,692	974	430	1,155	32	530	1,717	65	0	79	144
2000	81,165	36,522	10,749	981	427	1,161	32	489	1,683	90	1	135	226
2001	83,144	36,998	10,653	987	420	1,151	33	457	1,640	131	1	180	312
2002	85,172	37,338	10,456	995	410	1,129	33	421	1,583	183	2	231	416
2003	87,249	37,502	10,073	990	402	1,088	33	417	1,538	256	3	251	510
2004	89,377	37,458	9,705	977	391	1,048	32	397	1,477	329	4	288	621
2005	91,558	37,196	9,357	965	379	1,011	32	363	1,405	400	6	338	744
2006	93,928	36,779	8,684	951	369	938	31	373	1,342	509	7	349	864
2007	96,360	36,093	8,111	931	358	876	31	349	1,256	608	9	390	1,007
2008	98,857	35,085	7,539	910	345	814	30	327	1,171	708	10	431	1,149
2009	101,419	33,811	6,564	885	330	709	29	285	1,024	852	12	492	1,356
2010	104,050	32,246	5,836	854	310	630	28	234	892	971	14	563	1,549
2011	106,750	30,355	5,157	825	290	557	27	206	790	1,086	16	611	1,713
2012	109,521	28,244	4,417	797	271	477	26	189	692	1,208	18	648	1,875
2013	112,366	25,979	3,724	762	254	402	25	188	615	1,327	21	670	2,018
2014	115,287	23,591	3,074	724	237	332	24	165	521	1,442	23	715	2,179
2015	118,285	21,115	2,478	690	220	268	23	145	435	1,552	25	757	2,334
**Subtotal 1995**–**2010**						**16,289**	**498**	**6,827**	**23,614**	**5,110**	**70**	**3,748**	**8,929**
**Total 1995**–**2015**						**18,325**	**623**	**7,719**	**26,667**	**11,724**	**174**	**7,149**	**19,048**

a Infection defined as presence of at least one adult female worm.

Overall, we estimated that APOC has cumulatively averted 8.9 million DALYs due to onchocerciasis through 2010, and will avert another 10.1 million DALYs between 2011 and 2015, adding up to a total of 19.0 million DALYs averted through 2015. These updated estimates do not differ much from previous estimates (8.2 million DALYs averted through 2010, and another 9.2 million between 2011 and 2025). In relative terms, the burden of onchocerciasis in APOC areas has decreased from 23.1 DALYs per 1,000 persons in 1995 to 8.6 DALYs per 1,000 persons in 2010, and is expected to further decrease to 3.7 DALYs per 1,000 persons in 2015.

The updated disability weights provided by the GBD 2010 study are based on population surveys rather than expert opinion. Therefore, they are presumably less subjective and should better reflect our ideas and beliefs as a society of what constitutes health than previous disability weights [5]. However, it has been argued that the disability weights for visual impairment and blindness underestimate the burden of vision loss in rural Africa [9], [10]. One of the main arguments is that the surveys used to establish new disability weights did not adequately cover rural Africa (Tanzania only). Furthermore, being strictly a metric of health loss rather than wellbeing [5], DALYs do not capture the effects of vision loss and skin disease on socioeconomic status [11] and productivity [12], [13]. Therefore, the impact of APOC most likely encompasses more than what we report here in terms of health impact.

According to our updated estimates, skin disease is now the most important contributor to the burden of onchocerciasis, rather than eye disease. Moreover, the true disease burden of onchocercal skin disease (and the burden averted by APOC) is still larger than we estimate here, as our updated estimates do not include disfiguring skin disease, or other sequelae potentially associated with onchocerciasis, such as epilepsy [14] and head-nodding syndrome [15]. The additional burden of disfiguring skin disease is probably considerable, given the relatively high values of the updated disability weights for disfiguring skin disease and the high precontrol prevalence of disfiguring skin disease in areas endemic for onchocerciasis [6]. This underlines the importance of onchocercal skin disease, especially in forest areas where vision loss is relatively rare [16].
